# Biofilm formation and inflammatory potential of *Staphylococcus saccharolyticus:* A possible cause of orthopedic implant-associated infections

**DOI:** 10.3389/fmicb.2022.1070201

**Published:** 2022-11-28

**Authors:** Mastaneh Afshar, Andreas Møllebjerg, Gabriel Antonio Minero, Jacqueline Hollensteiner, Anja Poehlein, Axel Himmelbach, Jeppe Lange, Rikke Louise Meyer, Holger Brüggemann

**Affiliations:** ^1^Department of Biomedicine, Aarhus University, Aarhus, Denmark; ^2^Interdisciplinary Nanoscience Center (iNANO), Aarhus University, Aarhus, Denmark; ^3^Department of Genomic and Applied Microbiology, Institute of Microbiology and Genetics, University of Göttingen, Göttingen, Germany; ^4^Leibniz Institute of Plant Genetics and Crop Plant Research (IPK), Gatersleben, Germany; ^5^Department of Clinical Medicine, Aarhus University, Aarhus, Denmark; ^6^Department of Orthopedic Surgery Horsens Regional Hospital, Horsens, Denmark; ^7^Department of Biology, Aarhus University, Aarhus, Denmark

**Keywords:** *Staphylococcus saccharolyticus*, coagulase-negative staphylococci, anaerobes, biofilm, transcriptome, inflammation, implant-associated infection

## Abstract

*Staphylococcus saccharolyticus,* a coagulase-negative staphylococcal species, has some unusual characteristics for human-associated staphylococci, such as slow growth and its preference for anoxic culture conditions. This species is a relatively abundant member of the human skin microbiota, but its microbiological properties, as well as the pathogenic potential, have scarcely been investigated so far, despite being occasionally isolated from different types of infections including orthopedic implant-associated infections. Here, we investigated the growth and biofilm properties of clinical isolates of *S. saccharolyticus* and determined host cell responses. Growth assessments in anoxic and oxic conditions revealed strain-dependent outcomes, as some strains can also grow aerobically. All tested strains of *S. saccharolyticus* were able to form biofilm in a microtiter plate assay. Strain-dependent differences were determined by optical coherence tomography, revealing that medium supplementation with glucose and sodium chloride enhanced biofilm formation. Visualization of the biofilm by confocal laser scanning microscopy revealed the role of extracellular DNA in the biofilm structure. In addition to attached biofilms, *S. saccharolyticus* also formed bacterial aggregates at an early stage of growth. Transcriptome analysis of biofilm-grown versus planktonic cells revealed a set of upregulated genes in biofilm-embedded cells, including factors involved in adhesion, colonization, and competition such as epidermin, type I toxin-antitoxin system, and phenol-soluble modulins (beta and epsilon). To investigate consequences for the host after encountering *S. saccharolyticus*, cytokine profiling and host cell viability were assessed by infection experiments with differentiated THP-1 cells. The microorganism strongly triggered the secretion of the tested pro-inflammatory cyto- and chemokines IL-6, IL-8, and TNF-alpha, determined at 24 h post-infection. *S. saccharolyticus* was less cytotoxic than *Staphylococcus aureus*. Taken together, the results indicate that *S. saccharolyticus* has substantial pathogenic potential. Thus, it can be a potential cause of orthopedic implant-associated infections and other types of deep-seated infections.

## Introduction

Orthopedic implant-associated infections (OIAIs) are serious complications in orthopedic surgery and are associated with substantial morbidity and mortality (Ong et al., 2009; Arciola et al., 2015, 2018). Diagnosing and controlling OIAIs are challenging and costly (Osmon et al., 2013; Zimmerli, 2014). OIAIs often originate from the skin microbiota of the patient (Zimmerli, 2014; Månsson et al., 2021); skin-resident staphylococci, in particular *Staphylococcus aureus,* are the main causes of acute infections (Campoccia et al., 2006; Arciola et al., 2018; Oliveira et al., 2018). Delayed (3–10 weeks after surgery) or chronic (≥10 weeks) OIAIs are classically caused by bacteria with low virulence such as coagulase-negative staphylococci (CoNS), in particular *Staphylococcus epidermidis* (Zimmerli, 2014). Several different CoNS species have been described as causative agents of OIAIs (Zimmerli, 2014; Cuérel et al., 2017). Although CoNS species are considered less pathogenic than *S. aureus*, the continuous findings on properties of CoNS species, subspecies, and lineages have revealed a very heterogeneous group of bacteria, ranging from nonpathogenic to facultative pathogenic strains, with individual virulence potentials (Rosenstein and Götz, 2013).

A slow-growing CoNS species with a preference for anoxic growth conditions is *Staphylococcus saccharolyticus* (Evans and Hallam, 1978; Evans and Mattern, 1978). This species has recently been determined as the third most abundant CoNS species on human skin (Ahle et al., 2020, 2022), but its role on normal human skin is unknown. Also currently unknown is the frequency of *S. saccharolyticus*-caused or -associated infections, although some clinical cases have previously been reported, such as pyomyositis, bone marrow infections, endocarditis, spondylodiscitis, pneumonia, low-grade infections of the shoulder and a hospital outbreak of bacteremia (Steinbrueckner et al., 2001; Godreuil et al., 2005; Wu et al., 2009; Schneeberger et al., 2012; Liu et al., 2015; Young and Bhally, 2017). There are also some case reports of foreign body-related infections due to *S. saccharolyticus* (Oberbach et al., 2019; Söderquist et al., 2021). A recent study described seven cases of prosthetic hip and shoulder joint implant-associated infections associated with *S. saccharolyticus* (Söderquist et al., 2021).

Due to its slow and fastidious growth, isolation of *S. saccharolyticus* from clinical samples is often challenging (Brüggemann et al., 2019; Söderquist et al., 2021). Besides, *S. saccharolyticus* might be easily outcompeted by fast-growing species such as *S. epidermidis* on standard growth media. This could be one of the reasons why *S. saccharolyticus* remained overlooked in culture-dependent studies (Ahle et al., 2020, 2022). Regarding culture-independent studies, the lack of sufficient sequence differences in the 16S rRNA gene compared to other CoNS species and the lack of available reference genomes of *S. saccharolyticus* before 2019 caused frequent misidentifications (Brüggemann et al., 2019). Genome sequencing of 19 strains of *S. saccharolyticus* has been accomplished in the last 2 years (status: September 2022), revealing that the population of *S. saccharolyticus* is divided into two subclades, designated subclade 1 and subclade 2 (Brüggemann et al., 2019).

Despite its association with various infections, the pathogenicity of *S. saccharolyticus* remains largely unknown. In order to assess the pathogenic potential of this organism, more investigations are required. Thus, the aim of this study was to shed light on the microbial and host-interacting properties of *S. saccharolyticus*. Biofilm formation was monitored with different assays, revealing the ability of *S. saccharolyticus* to form distinct biofilms, and transcriptome sequencing of biofilm-grown and planktonic cells revealed the identity of biofilm-related genes. In addition, the inflammatory potential of *S. saccharolyticus* was monitored in cell culture experiments, revealing a profound induction of pro-inflammatory cytokines in macrophage-like cells. Taken together, the results of this study suggest that *S. saccharolyticus* has substantial pathogenic potential and can be a likely cause of OIAIs and, possibly, other types of deep-seated infections.

## Materials and methods

### Bacterial strains and growth conditions

Six clinical strains of *S. saccharolyticus* were used in this study (Table 1). They belong to the two distinct lineages of the species, designated subclades 1 and 2 (Brüggemann et al., 2019). These strains were previously isolated from blood cultures or OIAIs of patients at the Örebro University Hospital, Sweden (Söderquist et al., 2021). For transcriptome analyses, strain 13 T0028 was selected, due to the availability of its closed genome sequence. In cell culture infection experiments, two strains were selected: 13 T0028 (subclade 1) and DVP5-16-4677 (subclade 2). All strains were cultured on Fastidious Anaerobic Agar (FAA) plates (LAB M, Bury, United Kingdom), supplemented with 5% horse blood (v/v), and incubated at 37°C in anoxic conditions for 3 to 5 days. For liquid culture, brain-heart infusion-yeast extract broth supplemented with 0.05% (w/vol) cysteine (BHCY broth) was used as the base medium. Oxic and anoxic (Oxoid AnaeroGen System; Thermo Fisher Scientific, Waltham, MA, United States) growth conditions were applied. As control strains, *S. aureus* ATCC25923 and *S. epidermidis* strain 1457 were used (Supplementary Table S1).

**Table 1:** Information about the *S. saccharolyticus* strains used in this study.

Strain	Subclade	Description	Genbank accession number
12B0021	1	Clinical isolate from blood	HKG00000000
13 T0028	1	Clinical isolate from biopsy of PJI shoulder	CP068029-CP068030
05B0362	1	Clinical isolate from blood	QHKH00000000
DVP2-17-2406	2	Clinical isolate from biopsy of PJI hip	QHKD00000000
DVP4-17-2404	2	Clinical isolate from biopsy of PJI hip	QHKC00000000
DVP5-16-4677	2	Clinical isolate from biopsy of PJI hip	CP068031-CP068032

### Preparation of biofilms

Bacterial cultures were plated on FAA agar plates and incubated for 72 h at 37°C under anoxic conditions. A preculture was prepared in BHCY broth and the main culture in BHCY broth with or without the addition of supplements [1% glucose, 1% NaCl, 10 and 20% (v/v) human plasma] and incubated for 48 h at 37°C under anoxic conditions. Heparin-stabilized pooled human plasma was obtained from healthy donors at Aarhus University Hospital, Denmark. The OD_600_ of the culture medium was adjusted to 0.5, corresponding to a colony-forming unit (CFU) count of approx. 1×10^8^ per mL. For allowing initial bacterial adhesion, 200 μl of the culture was transferred to a polystyrene 96-well plate (Nunc^™^ MicroWell^™^ 96-Well, black) and incubated for 2 h at 37°C, with shaking (50 rpm) under anoxic conditions. The media was then replaced with 200 μl fresh media and biofilms were further grown anerobically for 48 h at 37°C, followed by another media exchange and 48 h incubation under the same conditions. Subsequently, biofilms were washed gently three times with phosphate-buffered saline (PBS). The experiments were repeated in three biological replicates.

### Biofilm imaging and volume calculation by optical coherence tomography

Biofilms were grown according to the above-described protocol. Subsequently, wells were filled with 200 μl of sterile PBS after the last washing step. Imaging of the biofilms was done by Optical Coherence Tomography (OCT) using an SD-OCT Ganymede 620C1 (Thorlabs GmbH, Dachau, Germany) with a central wavelength of 910 nm. Volume scans of 6 × 6 mm were recorded with a voxel size of 12 × 2 × 1.45 μm using an A-scan rate of 100 kHz. As a negative control, wells filled with sterile PBS were used. Experiments were performed in triplicates. The biofilm thickness was calculated from the two-dimensional cross-section images by a custom-written script. The images were passed through a median filter before segmentation. The images were segmented according to pixel intensity, with low-intensity pixels belonging to the background, high-intensity pixels belonging to the plastic surface, and medium intensity pixels belonging to the biofilm. Medium-intensity pixels below the plastic surface were removed by a custom-made filter. The biofilm thickness was calculated by multiplying the axial pixel count with the axial resolution. The mean biofilm thickness was calculated from the thickness of all points over the scanned area.

### Biofilm imaging by confocal laser scanning microscopy

Biofilms were grown according to the above-described protocol in flat-bottom 96-well plates (μ-plate 96-well, hydrophobic untreated, IBIDI), with or without the addition of 1% glucose and 1% NaCl to the media. Biofilms were gently washed three times with PBS and stained with 20 μM SYTO60 (Thermo Fisher Scientific, S11342) for live cells, and 10 μM TOTO-1 (Thermo Fisher Scientific, T3600) for visualizing dead cells and extracellular DNA (eDNA). Images of biofilms were taken by confocal laser scanning microscopy (CLSM; LSM700, Zeiss) using Plan-Apochromat 63x/1.40 NA objective, 54 μm pinhole, and excitation at 639 nm for SYTO60 (red) and 488 nm for TOTO-1 (green). The experiment was done in triplicates, and three images from each well were taken.

### Autoaggregation assay

Autoaggregation analysis was performed by a sedimentation assay as well as macroscopic and microscopic analyses. The sedimentation assay was performed as previously described (Hasman et al., 1999; Glaubman et al., 2016). In brief, 10^6^ CFU/ml of bacterial cells were suspended in two bottles of BHCY broth and incubated statically under anoxic conditions. The final optical density (OD_final_), determined at 600 nm, was assessed at the top of the culture tube after incubation at designated time points (2, 6, 10, 24, 48, 72 h). To determine the initial OD (OD_initial_), the same measurement was done for the other bottle with vortexing for 30 s before each time point. The turbidity reduction at the top of the culture is given as a percentage of the initial OD [100 × (OD_final_/OD_initial_)]. The experiments were performed for *S. saccharolyticus* 13 T0028 and *S. epidermidis* 1457. All experiments were performed in triplicates.

### *In vitro* cell culture infection model

The human leukemia monocytic cell line THP-1 (ATCC^®^TIB-202) was cultured in RPMI-1640/L-glutamine (Biowest) enriched with 10% fetal calf serum (Thermo Fisher Scientific) and supplemented with penicillin (100 U/ml) and streptomycin (100 μg/ml) in a humidified environment at 5% CO_2_ at 37°C. THP-1 monocytes were differentiated into macrophages by using 160 nM phorbol 12-myristate 13-acetate (PMA; Sigma Aldrich, Darmstadt, Germany) for 48 h on 0.24 × 10^6^ cells. *S. saccharolyticus* strains DVP5-16-4677 and 13 T0028, and *S. aureus* ATCC 25923 were cultured in BHCY broth to the mid-log growth phase. The bacteria were collected by centrifugation for 6 min at 5000 rpm, washed in sterile RPMI, resuspended, and diluted in RPMI to an OD_600_ of 0.5. For preparing heat-killed bacteria, the bacterial suspensions were adjusted to 10^8^ CFU/ml; the bacterial suspensions were heat-killed at 90°C for 3 h. Two MOIs (multiplicity of infection) were used: MOI 10 and MOI 100. As a positive control, 10^7^ HKLM/mL (Heat-killed *Listeria monocytogenes*, Invivogen) was used.

### Cytokine profiling

Supernatants from the cell culture infection experiments were collected after infection for 24 h and analyzed for the presence of pro-inflammatory cytokines. Measurements of IL-8, IL-6, and TNF-alpha were determined with IL-8 (ab214030, Abcam), IL-6 (ab178013, Abcam), and TNF-alpha (ab181421, Abcam) ELISA kits, following the manufacturer’s protocol.

### Cell viability

Cell viability was evaluated using a colorimetric cell-counting assay (WST-8/CCK8; ab228554, Abcam) according to the manufacturer’s instructions. Prior to its use, to avoid interferences due to bacterial activity, THP-1 cells were washed three times with PBS, and lysostaphin (1 U) was added and incubated for 15 min to kill the remaining extracellular bacteria. The level of produced formazan dye, measured by the absorbance (A) at 450 nm, is proportional to the number of metabolically viable cells. The percentage of viable cells was calculated as follows: % viability = [(A _test_ – A _background_)/(A _control_ – A _background_)] × 100.

### RNA extraction, sequencing, and transcriptome analysis

*Staphylococcus saccharolyticus* biofilm was obtained as described above. Planktonic cells were harvested after 2 h and 48 h of growth in microtiter plate wells (first and second step of media exchange). Biofilm-embedded cells were collected at 48 h of growth. The time point 48 h was chosen for comparative analysis between biofilm-embedded and planktonic cells. Harvested bacteria were resuspended in 800 μl RLT buffer (RNeasy Mini Kit, Qiagen) with β-mercaptoethanol (10 μl/ml) and cell lysis was performed using a laboratory ball mill. Subsequently, 400 μl buffer RLT (RNeasy Mini Kit Qiagen) with β-mercaptoethanol (10 μl/ml) and 1,200 μl 96% [v/v] ethanol were added. For RNA isolation, the RNeasy Mini Kit (Qiagen) was used, following the instructions of the manufacturer, but instead of buffer RW1, the buffer RWT (Qiagen) was used in order to also isolate RNAs smaller than 200 nt. To determine the RNA integrity number (RIN) the isolated RNA was run on an Agilent Bioanalyzer 2100 using an Agilent RNA 6000 Nano Kit, as recommended by the manufacturer (Agilent Technologies, Waldbronn, Germany). The remaining genomic DNA was removed by digestion with TURBO DNase (Invitrogen, Thermo Fisher Scientific, Paisley, United Kingdom). The Illumina Ribo-Zero plus rRNA Depletion Kit (Illumina Inc., San Diego, CA, United States) was used to reduce the amount of rRNA-derived sequences.

For sequencing, strand-specific cDNA libraries were constructed with a NEBNext Ultra II directional RNA library preparation kit for Illumina and the NEBNext Multiplex Oligos for Illumina (New England BioLabs, Frankfurt am Main, Germany). To assess the quality and size of the libraries, samples were run on an Agilent Bioanalyzer 2100 using an Agilent High Sensitivity DNA Kit, as recommended by the manufacturer (Agilent Technologies, Waldbronn, Germany). The concentration of the libraries was determined using the Qubit^®^ dsDNA HS Assay Kit, as recommended by the manufacturer (Life Technologies GmbH, Darmstadt, Germany). Sequencing was performed on a NovaSeq 6000 instrument (Illumina Inc., San Diego, CA, United States) using NovaSeq 6000 SP Reagent Kit v1.5 (100 cycles) and the NovaSeq XP 2-Lane Kit v1.5 for sequencing in the paired-end mode and running 2 × 50 cycles. For quality filtering and removing of remaining adaptor sequences, Trimmomatic-0.39 (Bolger et al., 2014) and a cutoff phred-33 score of 15 was used. Mapping against the reference genome was performed with Salmon (v 1.5.2; Patro et al., 2017). As mapping backbone, a file that contained all annotated transcripts excluding rRNA genes and the whole genome sequence of the reference as a decoy was prepared with a k-mer size of 11. Decoy-aware mapping was done in selective-alignment mode with “–mimicBT2,” “–disableChainingHeuristic,” and “–recoverOrphans” flags as well as sequence and position bias correction. For –fldMean and –fldSD, values of 325 and 25 were used, respectively. The quant. sf files produced by Salmon were subsequently loaded into R (v 4.0.3) using the tximport package (v 1.18.0; Soneson et al., 2015). DeSeq2 (v 1.30.0; Love et al., 2014) was used for normalization of the reads; fold-change shrinkages were also calculated with DeSeq2 and the apeglm package (v 1.12.0; Zhu et al., 2019). Genes with a log2-fold change of + 2/− 2 and a p-adjust value < 0.05 were considered differentially expressed.

### Statistics

Data were expressed as means ± standard deviations. Statistical analyses were conducted using the 2-tailed unpaired t-test for groups of two and ANOVA for multiple groups. *p*-values ≤0.05 were considered statistically significant (Welch’s *t*-test). The analyses were performed using GraphPad Prism 9.3.0 (Graph Pad Software).

## Results

### Some but not all *Staphylococcus saccharolyticus* strains can grow under oxic conditions

**Figure 1:**
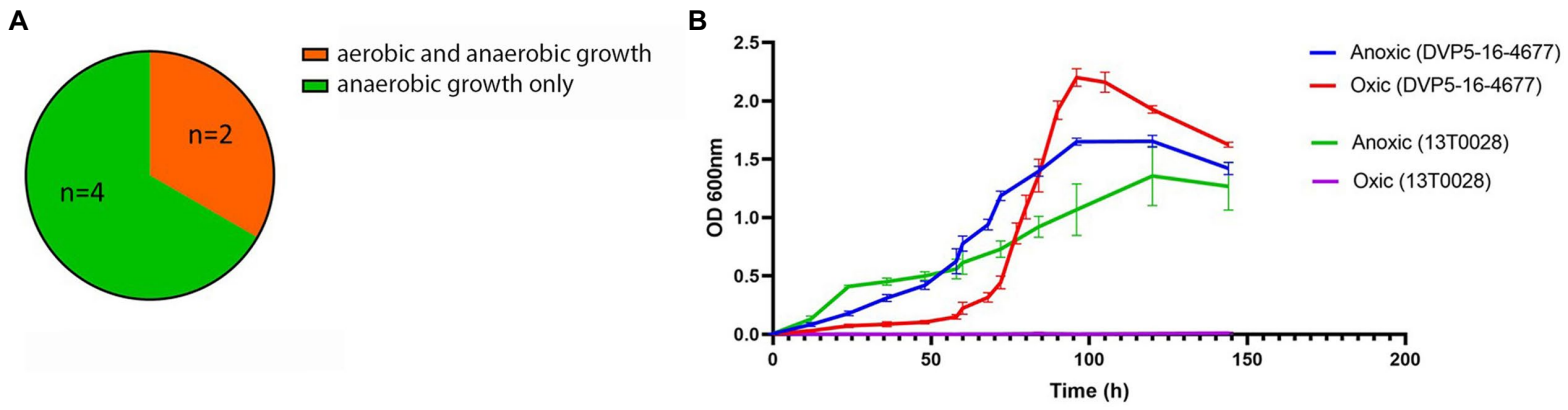
Growth kinetics of *S. saccharolyticus* in the presence and absence of oxygen. **(A)** Illustration of results of growth experiments under oxic and anoxic conditions of six different strains of *S. saccharolyticus.* **(B)** The growth curves of two strains of *S. saccharolyticus* (13 T0028, subclade 1; DVP5-16-4677, subclade 2) grown under oxic and anoxic conditions are depicted. The data are representative of three independent experiments.

Previous genome analyses have revealed that *S. saccharolyticus* strains can be divided into two phylogenetically distinct clades, designated subclade 1 and subclade 2 (Brüggemann et al., 2019). In order to test if different strains of *S. saccharolyticus* have similar growth properties, six strains, three of each subclade, were selected for cultivation in the presence and absence of oxygen. Cultivation of these six strains in BHCY broth showed that *S. saccharolyticus* strains demonstrated different growth patterns under oxic and anoxic conditions. All strains were able to grow under anoxic conditions with comparable growth kinetics. Under oxic conditions, however, only two strains, 05B0362 (subclade 1) and DVP5-16-4677 (subclade 2) demonstrated growth after 144 h of cultivation (Figure 1A). The growth curves of strain DVP5-16-4677 and strain 13 T0028 are shown in Figure 1B. Under oxic conditions, growth of strain DVP5-16-4677 is characterized by a longer lag phase, a steep log phase, and an increased growth yield compared to growth under anoxic conditions. This suggests substantial strain-specific, but not subclade-specific growth differences of *S. saccharolyticus* when cultivated under oxic conditions.

### Biofilm is formed by all tested *Staphylococcus saccharolyticus* strains and is stimulated by glucose and NaCl

**Figure 2:**
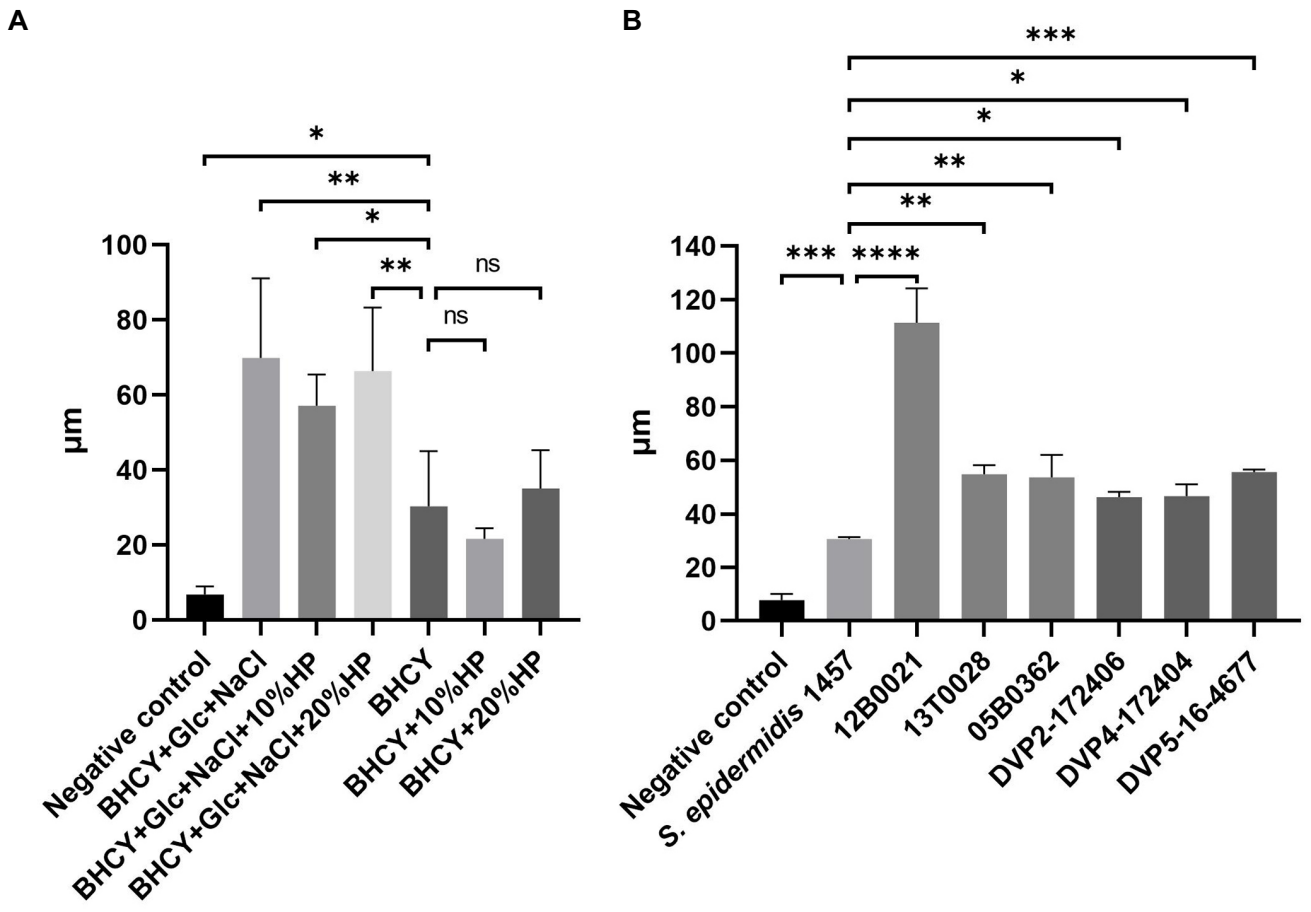
Biofilm formation of *S. saccharolyticus* quantified by OCT. **(A)** Biofilm formation of *S. saccharolyticus* strain 13 T00328 in culture media containing different supplements. Biofilm thickness was highest in BHCY broth supplemented with 1% glucose (Glc) and 1% NaCl. The media supplementation with human plasma (HP) did not lead to an additional increase in biofilm thickness. **(B)** Biofilm formation of different strains of *S. saccharolyticus*. As growth medium, BHCY supplemented with 1% glucose and 1% NaCl was used. *S. epidermidis* 1457 was used as positive control, and medium without bacteria as negative control. Three biological replicates were performed. ∗, *p* < 0.05; ∗ ∗, *p* < 0.01; ∗ ∗ ∗, *p* < 0.001; ∗ ∗ ∗ ∗, *p* < 0.0001; ns, not significant; determined by comparison to negative control (Welch’s *t*-test).

The effect of different media supplements on biofilm formation of *S. saccharolyticus* strain 13 T0028 was first evaluated by optical coherence tomography to identify conditions that were optimal for biofilm formation. The supplements glucose (1%), sodium chloride (NaCl, 1%), and human plasma (HP, 10, and 20%) were used separately and in combination. *S. saccharolyticus* 13 T0028 formed biofilm in all supplemented medium compositions, but with significant differences (Figure 2A). The biofilm formed by *S. saccharolyticus* 13 T0028 was more than twice as large when grown in medium supplemented with glucose and NaCl compared to non-supplemented medium, or medium supplemented with HP alone.

Biofilm formation in BHCY broth supplemented with 1% glucose and 1% NaCl was then quantified for six *S. saccharolyticus* strains representing the two subclades. *S. epidermidis* 1457 was included as a biofilm-positive control, as this strain is known to produce the polysaccharide intercellular adhesin (PIA) and has previously been shown to be a strong biofilm former (Mack et al., 1992; Schommer et al., 2011). OCT imaging showed that all six *S. saccharolyticus* strains formed biofilm (Figure 2B; Supplementary Figure S1). The biofilms of *S. saccharolyticus* were between 1.5 to almost 4 times thicker than biofilms formed by *S. epidermidis* 1457 under anoxic culture conditions. Five strains showed a very similar thickness ranging from 46.3 ± 1.9 μm to 54.7 ± 3.4 μm, whereas strain 12B0021 biofilm was 91.4 ± 16.7 μm thick. There was no significant difference in the biofilm thickness produced by subclade 1 and 2 strains, respectively.

### Biofilm visualization by confocal laser scanning microscopy

**Figure 3:**
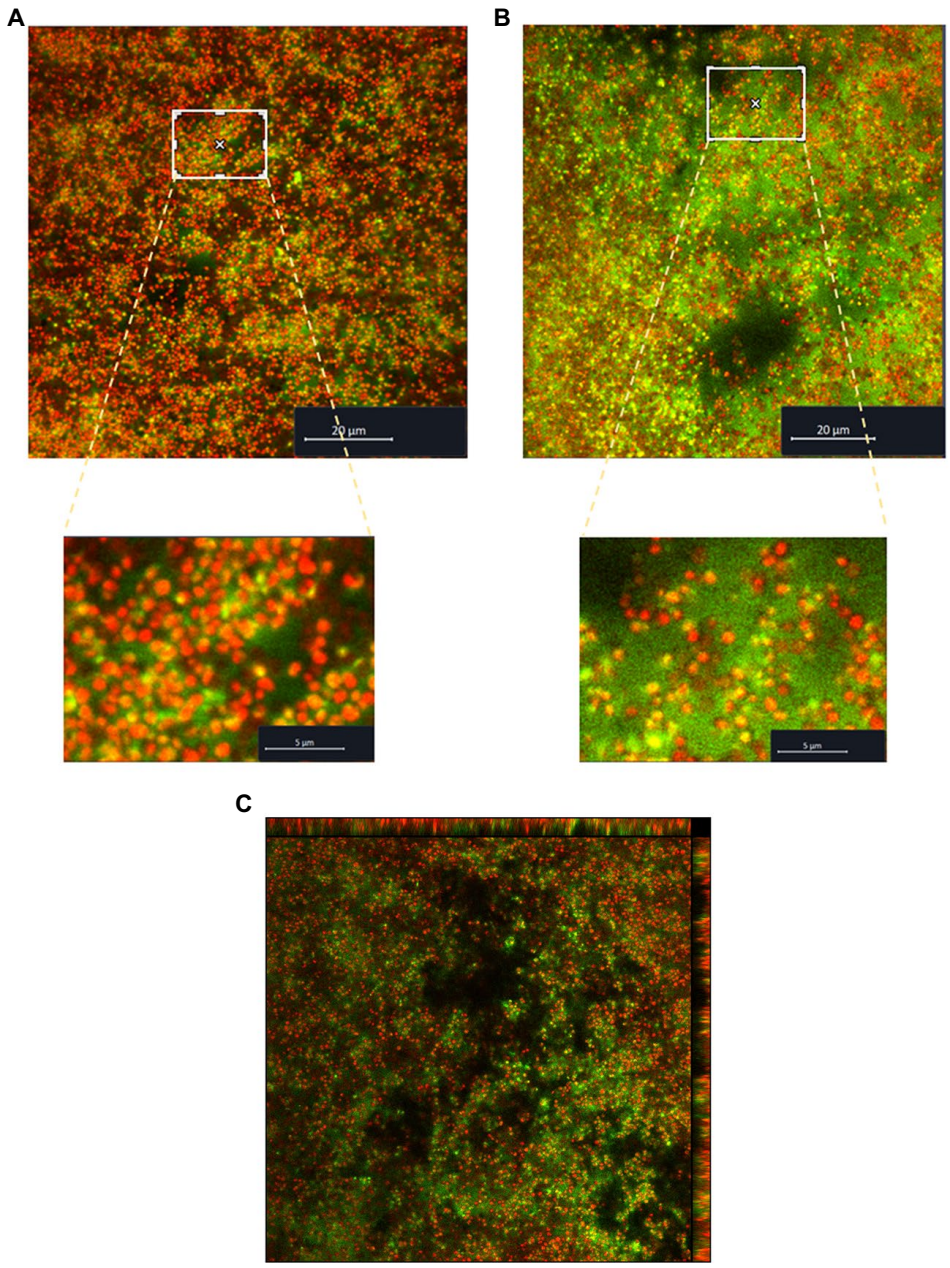
Visualization of the biofilm structure of *S. saccharolyticus* assessed by CLSM. **(A)** Biofilm structure formed by the strain 13 T0028 in BHCY supplemented with 1% glucose and 1% NaCl, visualized by CLSM. **(B)** Biofilm structure formed by strain 13 T0028 in BHCY without supplements. Viable cells were stained in red, and eDNA in green; dead cells appear in yellow (co-localization of red and green color). For better visualization, magnifications of selected regions are shown as zoom-in figures in **A** and **B**. **(C)** 3D view of the biofilm of *S. saccharolyticus*, grown in BHCY without supplements.

The biofilm formed by *S. saccharolyticus* 13 T0028 was visualized with CLSM by using SYTO60 to detect live bacterial cells and by using TOTO-1 for visualizing dead cells and eDNA (Figure 3). Image analyses indicated that lower amounts of eDNA were present in biofilms grown in media with glucose and NaCl supplementation (Figure 3A) compared to biofilms grown in media without supplementation (Figure 3B). The eDNA seemed to originate from cell lysis as eDNA was detected mostly around the coccus-shaped cells as judged from a 3D view of the biofilm (Figure 3C).

### *Staphylococcus saccharolyticus* has autoaggregation activity

**Figure 4:**
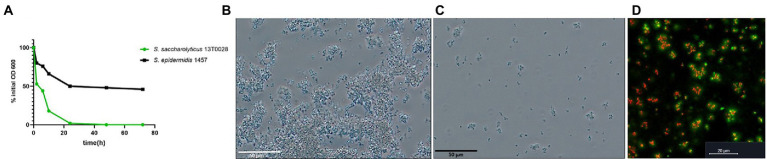
Autoaggreation of *S. saccharolyticus*. Autoaggregation of *S. saccharolyticus* 13 T0028 and *S. epidermidis* 1457 assessed by a sedimentation assay. Turbidity reduction is determined as a percentage of the initial OD_600_ value **(A)**. Microscopical analysis of autoaggregation in *S. saccharolyticus* 13 T0028 **(B)** and *S. epidermidis* 1457 **(C)**, respectively, grown to an OD_600_ of 0.5. Bacterial aggregates of viable cells (red) and dead cells (yellow) of *S. saccharolyticus* 13 T0028 and eDNA (green) visualized by CLSM **(D)**.

A sedimentation assay was performed to evaluate whether *S. saccharolyticus* is able to auto-aggregate. Sedimentation of *S. saccharolyticus* 13 T0028 was significantly more pronounced compared to *S. epidermidis* 1457 (Figure 4A). Microscopy analysis confirmed the presence of larger bacterial aggregates (>50 μm) of *S. saccharolyticus* 13 T0028 (Figure 4B) compared to *S. epidermidis* 1457 (Figure 4C) after the OD_600_ had decreased to 0.5. Autoaggregation could also be detected macroscopically (Supplementary Figure S2). CLSM imaging of the aggregates of *S. saccharolyticus* showed that eDNA was often detected in and around such aggregates (Figure 4D).

### Transcriptome analysis of *Staphylococcus saccharolyticus* grown in biofilm versus planktonic cells

**Figure 5:**
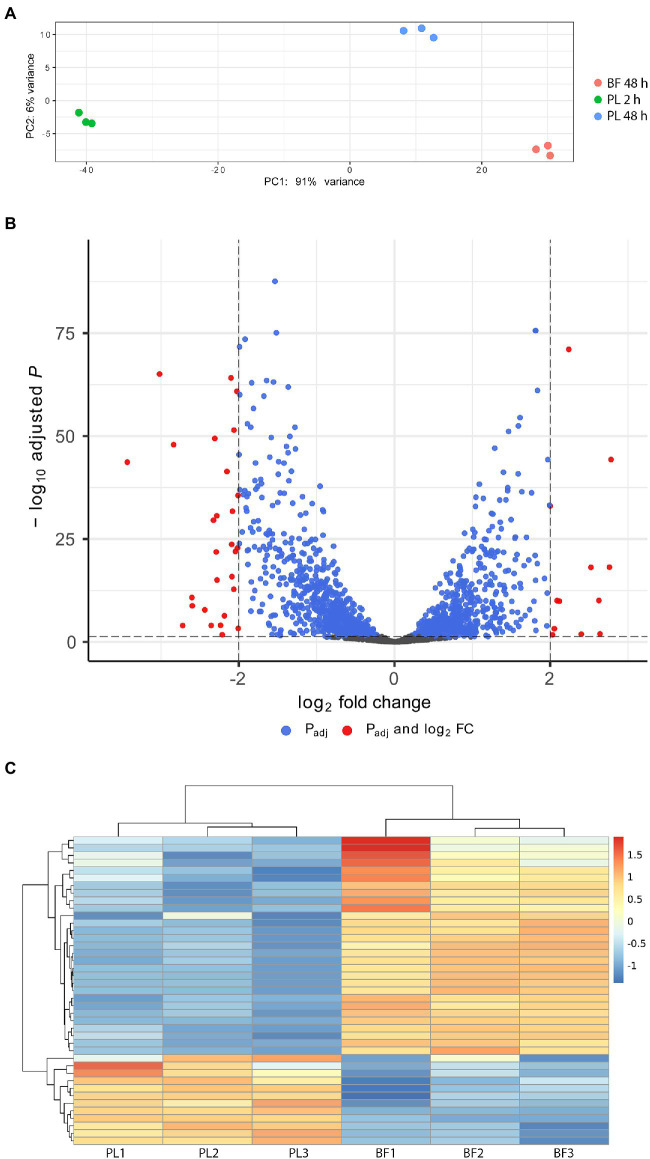
Comparative gene expression analysis between biofilm-embedded and planktonic cells of *S. saccharolyticus*. **(A)** Principal component analysis (PCA) of the gene expression data of nine cultures, including three applied conditions (biofilm at 48 h (BF 48 h), planktonic cells at 48 h (PL 48 h) and at 2 h (PL 2 h)) in three biological replicates. **(B)** Volcano plot of differentially expressed genes between biofilm and planktonic cells at 48 h. The plot shows the log2-fold-change values on the X-axis against the adjusted *p* values (−log10 scale) on the Y-axis. Red dots represent genes that showed gene expression differences with log2-fold change >2 or < −2. **(C)** Heatmap of differentially expressed genes between biofilm (BF) and planktonic (PL) cells at 48 h; shown is the data for all three biological replicates. The corresponding locus tags and gene annotations are listed in Table 2.

To explore the nature and mechanism of biofilm formation of *S. saccharolyticus,* we analyzed genome-wide gene expression using RNA sequencing. Biofilm-embedded cells of *S. saccharolyticus* strain 13 T0028 grown in BHCY supplemented with 1% glucose and 1% NaCl were harvested at 48 h of anaerobic growth. In addition, planktonic cells were also harvested at 48 h of growth and at an earlier time point (2 h). Genome-wide gene expression was analyzed in three biological replicates. A principle component analysis (PCA) plot showed that gene expression was substantially different between these three conditions (2 h and 48 h planktonic cells, 48 h biofilm cells; Figure 5A). We focused in subsequent analyses on the differential gene expression between biofilm-grown and planktonic cells harvested at 48 h. Applying of log2-fold-change cutoff of ≥2 and ≤ −2, in total, 27 and 11 genes were up- or downregulated, respectively, in biofilm-embedded cells compared to planktonic cells (Figures 5B,C; Table 2). For a log2-change cutoff of ≥1.5 and ≤ −1.5, in total, 104 and 51 genes were up- or downregulated, respectively (Supplementary Table S2). Among the upregulated genes in biofilm conditions were several genes encoding functions with potential relevance in bacterial interference/competition such as epidermin, type I toxin-antitoxin system, and phenol-soluble modulins (PSMs; Table 2; Supplementary Table S2). Interestingly, among the 11 downregulated genes, seven (63.6%) were frameshifted or fragmented and are likely not functional; among the 27 upregulated genes only six were frameshifted/fragmented (22.2%). Gene expression differences of known or suspected biofilm-relevant genes that are present also in other staphylococci were checked, including genes that encode for proteins involved in adhesion and exopolysaccharide production (Santos et al., 2022). The *icaADBC* genes, responsible for PIA production, are present in the genome of *S. saccharolyticus*; however, they are not expressed (*icaB*) or even downregulated (*icaD*) in biofilm-embedded cells (Table 2; Supplementary Table S3). Furthermore, the *ica* locus seems to be inactivated by frameshift mutations, as previously noted (Brüggemann et al., 2019). These two findings suggest that the mechanism of biofilm formation in *S. saccharolyticus* is PIA-independent. In contrast, the expression of a number of genes coding for cell wall-anchored (CWA) proteins was elevated in biofilm-embedded cells. Examples are cell wall-anchored proteins containing host attachment domains (microbial surface components recognizing adhesive matrix molecules, MSCRAMMs) such as SdrG and SdrH (fibrinogen-binding adhesins), ClfB (clumping factor), SasD, and covalently linked CWA proteins such as SDR family proteins (Supplementary Table S3). In addition, the slightly elevated gene expression of the autolysin gene *atl* and the elevated gene expression of the septum formation initiator protein (DMB76_010640) might indicate an enhanced release of eDNA as another factor in biofilm formation. As possible accessory systems involved in biofilm maturation, a set of genes are upregulated in biofilm-embedded cells, such as sortase, β-hemolysin, and β-PSMs that have a role in biofilm enhancement, maturation, and dissemination (Wang et al., 2011). These genes are likely under the control of quorum sensing; interestingly, the structural gene of the autoinducing peptide precursor, *agrD*, was also upregulated in biofilm-embedded compared to planktonic cells (Supplementary Table S3).

**Table 2:** List of differentially expressed genes of *S. saccharolyticus* 13 T0028 grown in biofilm compared to planktonic cells at 48 h with a log2-fold change of >2 or < −2.

Locus tag	Annotation	mean expression	log2-fold change
DMB76_004255	hypothetical protein	374.6	3.4
DMB76_009105	epsilon family phenol-soluble modulin	47.5	3.4
DMB76_001650	aspartate 1-decarboxylase	1268.5	3.0
DMB76_000275	hypothetical protein	8801.7	2.8
DMB76_011400	type I toxin-antitoxin system Fst family toxin	29.8	2.7
DMB76_011110	GlsB/YeaQ/YmgE family stress response membrane protein	8802.0	2.6
DMB76_000660	ABC transporter ATP-binding protein	33.8	2.6
DMB76_008440	gallidermin family lantibiotic	105.0	2.4
DMB76_010640	septum formation initiator family protein	742.6	2.3
DMB76_001580	LPXTG cell wall anchor domain-containing protein	472.1	2.3
DMB76_002575	hypothetical protein	10332.9	2.3
DMB76_000380	MptD family putative ECF transporter S component	163.7	2.3
DMB76_009220	DUF4887 domain-containing protein	3417.0	2.3
DMB76_004810	type I toxin-antitoxin system Fst family toxin	42.5	2.2
DMB76_001590	hypothetical protein	59.8	2.2
DMB76_001620	L-lactate dehydrogenase	59345.1	2.2
DMB76_001350	DegT/DnrJ/EryC1/StrS family aminotransferase	31127.7	2.1
DMB76_008990	MetQ/NlpA family ABC transporter substrate-binding protein	9714.4	2.1
DMB76_002130	hypothetical protein	1654.6	2.1
DMB76_005335	universal stress protein	6535.2	2.1
DMB76_001480	hypothetical protein	22065.1	2.1
DMB76_001355	LCP family protein	28412.8	2.1
DMB76_002790	SDR family oxidoreductase	198300.7	2.0
DMB76_001345	acetyltransferase	11672.2	2.0
DMB76_008980	CsbD family protein	280.0	2.0
DMB76_002785	amidohydrolase	97240.6	2.0
DMB76_001640	3-methyl-2-oxobutanoate hydroxymethyltransferase	8016.6	2.0
DMB76_007480	DNA-protecting protein DprA	37.7	−2.1
DMB76_008220	hypothetical protein	5.7	−2.1
DMB76_004380	DUF3267 domain-containing protein	104.6	−2.1
DMB76_002540	copper resistance protein CopC	94.3	−2.1
DMB76_006500	class I SAM-dependent RNA methyltransferase	1274.6	−2.2
DMB76_000990	intracellular adhesion protein D	7.0	−2.4
DMB76_009775	Bax inhibitor-1/YccA family protein	155.8	−2.5
DMB76_010290	deoxynucleoside kinase	172.0	−2.6
DMB76_001160	Csa1 family protein	5.7	−2.7
DMB76_006280	DUF309 domain-containing protein	119.9	−2.8
DMB76_010395	class I SAM-dependent methyltransferase	529.3	−2.8

### *Staphylococcus saccharolyticus* induces a pro-inflammatory response in macrophage-like cells

**Figure 6:**
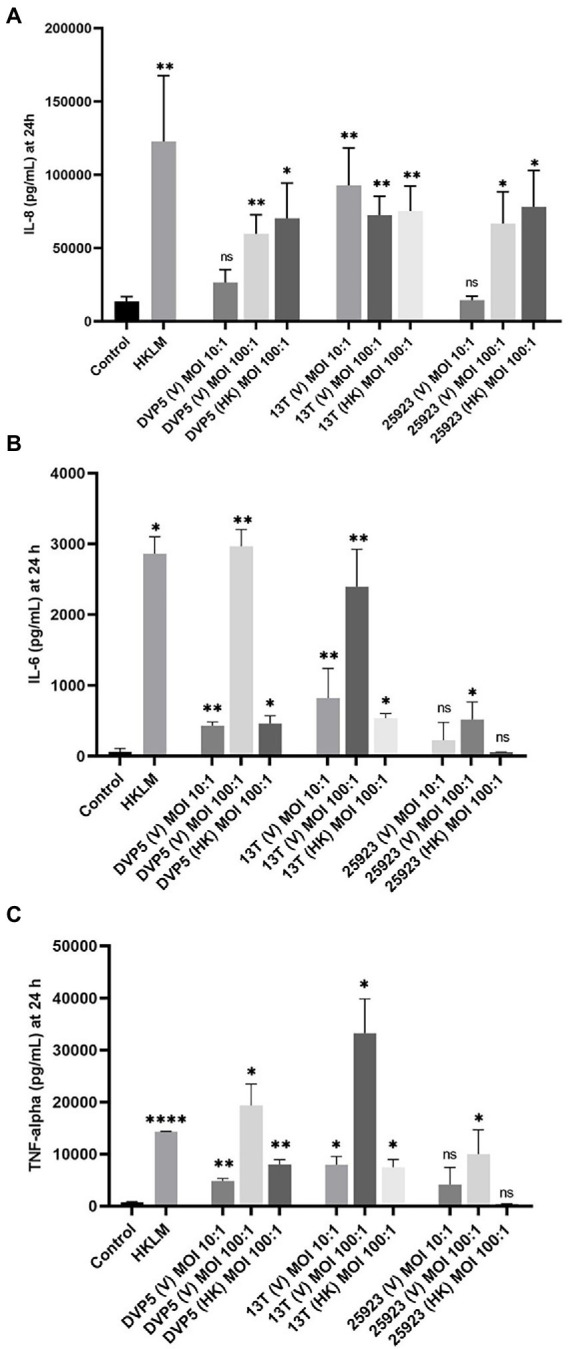
Pro-inflammatory chemo- and cytokine production in macrophage-like cells triggered by *S. saccharolyticus*. THP-1 cells were differentiated and exposed to the *S. saccharolyticus* strains DVP5-16-4677 (DVP5) and 13 T0028 (13 T) as well as to *S. aureus* ATCC 25923. Cell supernatants were harvested after 24 h and levels of the chemo- and cytokines IL-8 **(A)**, IL-6 **(B)**, TNF-alpha **(C)** were determined. Viable (V) bacteria were tested at two MOIs (10, 100) and heat-killed (HK) bacteria were tested at MOI 100. Heat-killed *Listeria monocytogenes* (HKLM) as a well-studied TLR-2 agonist was used as a positive control. Results are expressed as mean ± SEM, and significant differences compared to untreated cells are highlighted by asterisks (*, *p* < 0.05; **, *p* < 0.01; ****, *p* < 0.0001; ns, not significant; Welch’s *t*-test for parametric distribution data and Mann–Whitney test for non-parametric distribution data were used).

It has been shown in previous studies that staphylococci associated with OIAIs induce significant local increases in pro-inflammatory cytokine levels (Gollwitzer et al., 2013; Prince et al., 2020). In this study, levels of important pro-inflammatory cyto- and chemokines (IL-8, IL-6, and TNF-alpha) were determined in THP-1 cell culture experiments upon exposure to *S. saccharolyticus*. A strain of *S. aureus* (ATCC 25923) was examined along with the two *S. saccharolyticus* strains DVP5-16-4677 and 13 T0028 (one strain from each subclade). Both strains of *S. saccharolyticus* promoted the production and secretion of all three tested chemo- and cytokines, determined at 24 h post-infection, albeit with strain differences (Figure 6). IL-8 was triggered by all viable and heat-killed staphylococcal strains used (Figure 6A). At a multiplicity of infection (MOI) of 100, viable and heat-killed bacteria induced very similar levels of IL-8, with an induction of 4.5-7-fold compared to the negative control. Infection with *S. saccharolyticus* strain DVP5-16-4677 resulted in a dose-dependent profile of IL-8 induction, comparable with *S. aureus* ATCC 25923. In contrast, infection with *S. saccharolyticus* strain 13 T0028 triggered an enhanced IL-8 production already at an MOI of 10. A dose-dependent increase in cytokine levels was detected for both tested strains of *S. saccharolyticus* concerning the production of the two pro-inflammatory cytokines IL-6 and TNF-alpha (Figures 6B,C). For instance, at MOI 100, the strains DVP5-16-4677 and 13 T0028 induced a 46- and 37-fold increase, respectively, of IL-6 levels compared to the negative control (Figure 6B). In contrast, *S. aureus* ATCC25932 induced IL-6 levels only 8-fold. Interestingly, heat-killed strains of *S. saccharolyticus* only mildly induced IL-6 and TNF-alpha levels.

### *Staphylococcus saccharolyticus* is not cytotoxic to macrophage-like cells

**Figure 7:**
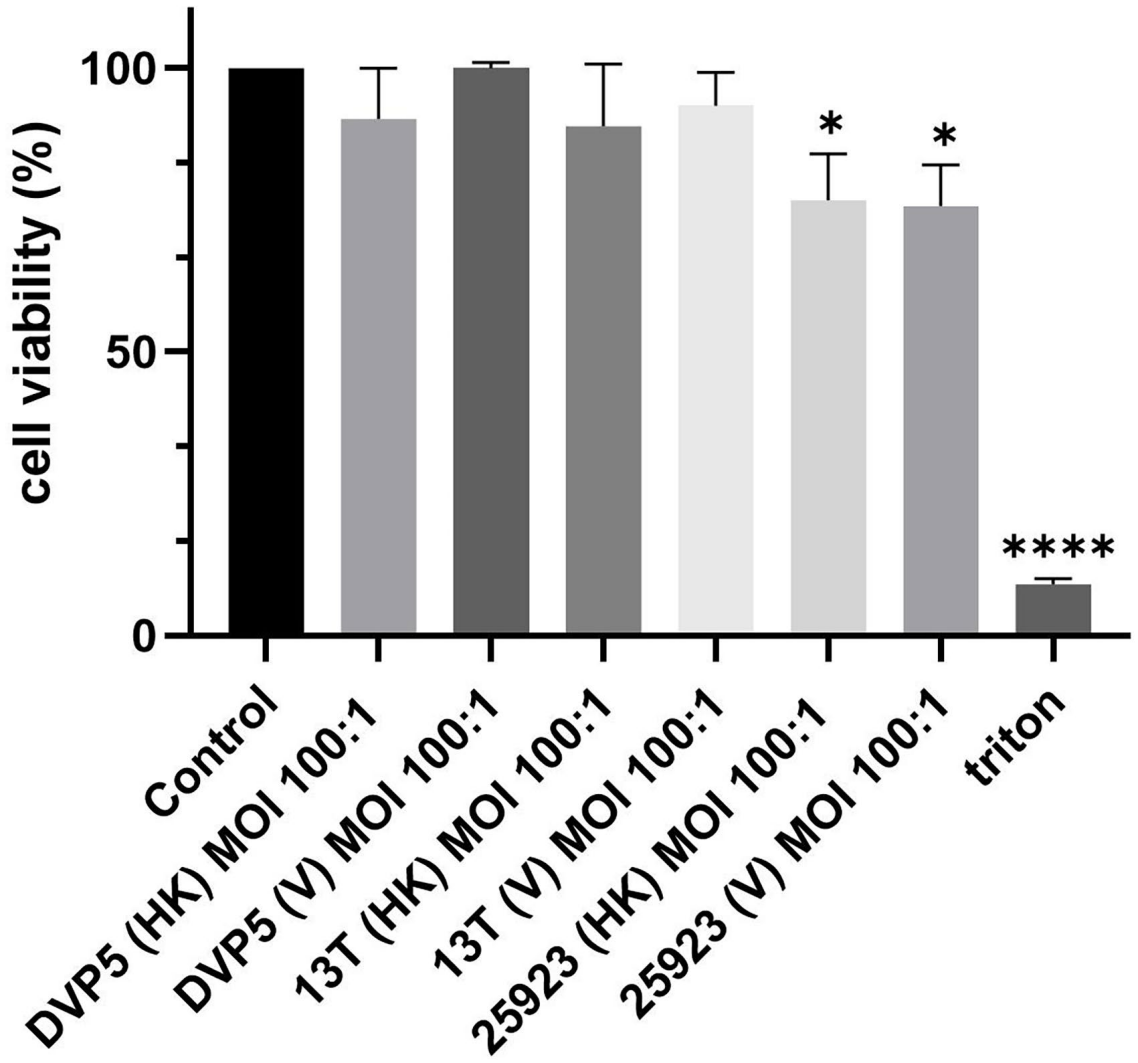
*S. saccharolyticus* has no cytotoxic effect on THP-1 cells. *S. saccharolyticus* does not induce cytotoxcity in THP-1 cells after 24 h of infection. A WST-8 assay was applied to differentiated THP-1 cells exposed to the following bacterial strains: *S. saccharolyticus* strains DVP5-16-4677 (DVP5; subclade 2) and 13 T0028 (13 T; subclade 1) and *S. aureus* ATCC 25923. Triton X-100 was used as positive control. HK, heat-killed; V, viable; MOI, multiplicity of infection. Two biological replicates were performed for each strain. Results are expressed as mean ± SEM, and significant differences compared to untreated cells are highlighted by asterisks (*, *p < 0.05*; ****, *p* < 0.0001; Welch’s *t*-test).

To examine if *S. saccharolyticus* has any impact on the host cell fate, the viability of THP-1 cells after bacterial exposure was assessed by a WST-8 assay. Exposure of host cells to *S. saccharolyticus* for 24 h did not cause statistically significant differences in cell viability between infected and non-infected cells with live and heat-killed bacteria (Figure 7). In contrast, *S. aureus* infection had a significant effect on cell viability (*p* < 0.05), indicative of cytotoxic activity.

## Discussion

Knowledge about *S. saccharolyticus* is scarce. A recent study showed that this CoNS species is much more abundant on human skin than previously anticipated; it may represent a significant portion of the normal skin microbiota, in particular on the skin of the upper back (Ahle et al., 2020, 2022). The organism is often overlooked in culture-dependent studies due to its anaerobic, fastidious growth properties that are unusual for a CoNS species (Evans and Hallam, 1978). Thus, it seems plausible that the species is also often overlooked in the clinical setting, since microbial identification often relies on cultivation. If correctly identified, it has previously often been considered a skin-derived contaminant (Trojani et al., 2020). In contrast, a few studies have described *S. saccharolyticus* as a potential cause of a series of human infections such as spondylodiscitis, pneumonia, endocarditis, and prosthetic joint infections (Westblom et al., 1990; Wu et al., 2009; Trojani et al., 2020; Wang et al., 2020).

To date, the potential pathogenicity of *S. saccharolyticus* and its interactions with the host have not been studied. Here, a study was performed using clinical strains of *S. saccharolyticus* that have been mainly isolated from OIAIs (Söderquist et al., 2021). The study aimed at the investigation of the pathogenic potential of this bacterium in order to evaluate if it could possibly be a causative agent of deep-seated infections. Our study revealed that clinical strains of *S. saccharolyticus* are able to produce biofilms. The tested strains also had strong inflammatory potential as judged from cell culture infection experiments. The study also highlighted that there are strain differences regarding the mentioned properties. A few results should be discussed in the following in more detail.

*Staphylococcus saccharolyticus* is a fastidious organism that depends on nutrient-rich media (Evans and Hallam, 1978); it has exclusively been isolated from anaerobically cultured samples so far. We investigated the growth properties of six *S. saccharolyticus* strains. Two out of six tested strains were capable of growing aerobically as well as anaerobically the reason for such strain differences regarding their growth properties is currently unknown. The genome of *S. saccharolyticus* contains over 400 pseudogenes, and strain differences exist regarding the total number of pseudogenes. It is possible that a gene that is essential for growth in oxic conditions is frameshifted in strains that solely rely on anoxic conditions. A first examination detected no obvious frameshift mutations or premature stop codons in the genes of the respiratory chain or oxygen detoxification systems, such as catalase and superoxide dismutase (Brüggemann et al., 2019).

We investigated biofilm formation of six *S. saccharolyticus* strains and found strong biofilm production under the tested conditions. The strongest level of biofilm production was seen in the presence of glucose and NaCl, which is in agreement with previous studies on other staphylococcal species (Lim et al., 2004; Agarwal and Jain, 2013). Transcriptomic analyses found genes specifically upregulated in biofilm-grown bacteria. The mechanisms of biofilm formation by some other staphylococci are relatively well characterized. One important mechanism is the production of extracellular polysaccharides, such as PNAG (poly-N-acetyl glucosamine), whose biosynthesis is under the control of enzymes encoded by the *ica* operon (Heilmann et al., 1996; Gerke et al., 1998; Jabbouri and Sadovskaya, 2010). The *ica* locus in the genome of *S. saccharolyticus* carries many frameshift mutations, and is thus most likely not functional (Brüggemann et al., 2019). Despite the importance of the *ica* operon, biofilm formation in staphylococci can also be *ica*-independent. The mechanisms that account for *ica*-independent biofilm formation among CoNS are manifold (Fitzpatrick et al., 2005; Büttner et al., 2015). For instance, specific factors such as the accumulation-associated protein (Aap) and other adhesins play key roles in the formation of *ica*-independent biofilms (Schaeffer et al., 2015; Paharik and Horswill, 2016). *S. saccharolyticus* has no homolog of Aap. However, gene expression data obtained here suggest that *S. saccharolyticus* is equipped with an array of other adhesins/MSCRAMMs. For instance, cell wall-anchored proteins are produced, such as SdrG, SdrH, and ClfB that interact specially with human extracellular matrix (ECM) proteins (Otto, 2009). In addition, the expression level of a gene responsible for autolysin production was also elevated in biofilm-embedded bacteria compared to planktonic cells. This protein has two characteristics that could stimulate biofilm formation: its attachment ability to ECM proteins, and its cell lysis activity which results in the release of eDNA, a well-known component of biofilm which promotes intercellular aggregation (Das et al., 2014). A significant role of eDNA in primary attachment of *S. epidermidis* was revealed in previous studies, as addition of DNase I could abolish bacterial attachment to glass surfaces (Qin et al., 2007). We could detect and visualize eDNA in *S. saccharolyticus* biofilms, in particular when the bacteria were grown in the absence of medium supplementation. However, the precise role of eDNA in *S. saccharolyticus* biofilms has to be clarified in a future study, including experiments with DNase treatment.

Staphylococcal biofilms can form on the surface of implants or tissues, but they can also form as non-adherent multicellular aggregates (Crosby et al., 2016; Otto, 2018; Schilcher and Horswill, 2020; Burel et al., 2021). Such aggregates have the same phenotypic properties as adherent biofilms in terms of immune evasion and antimicrobial tolerance. We showed the ability of *S. saccharolyticus* to form such aggregates that also contained eDNA.

Thus, *S. saccharolyticus* possesses fundamental properties that could enable the formation of persistent infections and aid the bacteria to evade immune clearance and tolerate antibiotics. The next question is if and how *S. saccharolyticus* is recognized by the host; in particular, how does the immune system respond to the presence of *S. saccharolyticus* in deeper tissue sites? The severity of OIAIs can strongly be influenced by specific interactions of the bacteria with the host immune system. In this study, we focused on macrophage responses to *S. saccharolyticus,* as one of the critical immune cell types in infectious diseases. The outcome of an infection is thought to be fundamentally reliant on the initial reaction of these decisive innate immune cell players (Amin Yavari et al., 2020). We determined the cytokine production levels upon exposure of THP-1 cells to *S. saccharolyticus* and found high production of IL-6, IL-8, and TNF-alpha, indicating that *S. saccharolyticus* is a strong pro-inflammatory stimulus, which at least partially depends on the viability of the bacterium. Interestingly, IL-6 and TNF-alpha production in THP-1 cells triggered by *S. aureus* is less pronounced compared with *S. saccharolyticus.* It is possible that the maximal levels of secreted cytokines were reached before 24 h post-infection and then gradually declined, which is consistent with the results of a previous study (Das et al., 2008). Another explanation could be that *S. aureus*, in contrast to *S. saccharolyticus*, has the ability to limit the production of pro-inflammatory cytokines produced by macrophages, as one of several mechanisms of this bacterium to dampen host immune responses (Thurlow et al., 2011; Peres et al., 2015; Ricciardi et al., 2018). This could imply that *S. saccharolyticus* infections are easier to trace by the host and could be more efficiently cleared by host immune cells compared with *S. aureus*.

In contrast to *S. aureus, S. saccharolyticus* does not have a significant effect on host cell viability. This could be explained with the lack of several cytolytic toxins in *S. saccharolyticus* that are produced by *S. aureus*, including α-hemolysin, leukocidins and PSMα (including δ-hemolysin) (Kitur et al., 2015). However, both organisms possess the gene for the beta-hemolysin (sphingomyelinase). Alternatively, the lack of cytotoxicity of *S. saccharolyticus* could also be explained with the used conditions, since cell culture experiments were carried out under aerobic conditions, where *S. saccharolyticus*, despite being aerotolerant, does not grow significantly during the infection time period, in contrast to *S. aureus*.

There are a number of limitations to our study. We only used one cell type in infection experiments. In addition, we only monitored the levels of three chemo−/cytokines at one specific time point after infection (24 h). Future studies are needed to determine and investigate the precise inflammatory potential of the microorganism, the involved host cell receptors and bacterial components that are responsible for the immunostimulatory activity. For instance, bacterial lipoproteins that are recognized by Toll-like receptor 2 have been identified to be important immunostimulatory factors in other staphylococci (Nguyen and Götz, 2016). Staphylococci have developed additional strategies to sustain within the host and influence the progression of an infection. *S. aureus* and CoNS are able to switch from an extracellular to an intracellular lifestyle, in order to escape or delay immune recognition, which might facilitate chronic infections (Ko et al., 2013; Heilmann et al., 2019; Moldovan and Fraunholz, 2019; Bogut and Magryś, 2021). Thus, it needs to be clarified in future studies if *S. saccharolyticus* can invade, persist and or/replicate in host cells. In addition, further studies are needed, including transcriptome, proteome, and metabolome studies, to understand and investigate the general metabolism of *S. saccharolyticus,* as well as its specific traits that allow successful tissue colonization and propagation within the host.

## Conclusion

In spite of previous case reports of *S. saccharolyticus* isolated from various human infections including OIAIs, it has not been possible yet to clearly link *S. saccharolyticus* to OIAIs and define its role in such infections. This study was performed to explore whether *S. saccharolyticus* has a pathogenic potential and thus assess if it could be a true causative agent of deep-seated infections. The results of this study revealed the biofilm-producing ability of *S. saccharolyticus* in attached and aggregated forms. The comparative transcriptome analysis discovered a set of differentially expressed genes in biofilm-embedded cells, including a number of adhesins. Based on the apparent inactivity of the *ica* operon, it is predicted that the biofilm formation in *S. saccharolyticus* relies on a PIA-independent strategy. Our study further highlighted strong pro-inflammatory responses in macrophages upon exposure to *S. saccharolyticus*. Taken together, our findings support the assumption that *S. saccharolyticus* can be a cause of deep-seated infections such as OIAIs.

## Data availability statement

The original contributions presented in the study are publicly available. This data can be found at: NCBI, PRJNA890184.

## Author contributions

MA, JL, RLM, and HB contributed to the conception and design of the study. MA performed wet lab benchwork and analyzed data. MA, AM, and GAM contributed to OCT and CSLM analyses. AP, JH, AH, and HB contributed to sequence data generation and analyses. MA and HB wrote the manuscript. All authors contributed to the article and approved the submitted version.

## Funding

This research was supported with funds from the Novo Nordisk Foundation (grant no. NNF18OC0053172) and the “Fabrikant Vilhelm Pedersen og Hustrus Legat” (by the recommendation from the Novo Nordisk Foundation).

## Conflict of interest

The authors declare that the research was conducted in the absence of any commercial or financial relationships that could be construed as a potential conflict of interest.

## Publisher’s note

All claims expressed in this article are solely those of the authors and do not necessarily represent those of their affiliated organizations, or those of the publisher, the editors and the reviewers. Any product that may be evaluated in this article, or claim that may be made by its manufacturer, is not guaranteed or endorsed by the publisher.
