# Supplementary material for: Biofilm formation and inflammatory potential of Staphylococcus saccharolyticus: A possible cause of orthopedic implant-associated infections
Source: Front Microbiol. 2022 Nov 28;13:1070201. doi: 10.3389/fmicb.2022.1070201 (PMC9742538; doi:10.3389/fmicb.2022.1070201)
Supplement: Supplementary file 1 [file Table_1.DOCX]

**Supplementary table S1**. Information about other staphylococcal strains used in this study

| Strain | Source /properties | Genbank Accession Number |
| --- | --- | --- |
| *S. aureus* ATCC 25923 | obtained from ATCC | NZ_CP009361 |
| *S. epidermidis* 1457 | central venous catheter infection/ biofilm producer, PIA-producing | CP020463.1 |
