# Supplementary material for: Biofilm formation and inflammatory potential of Staphylococcus saccharolyticus: A possible cause of orthopedic implant-associated infections
Source: Front Microbiol. 2022 Nov 28;13:1070201. doi: 10.3389/fmicb.2022.1070201 (PMC9742538; doi:10.3389/fmicb.2022.1070201)
Supplement: Supplementary file 3 [file Table_3.DOCX]

**Supplementary table S3**. Differential expression of selected biofilm-relevant genes in *S. saccharolyticus* 13T00028 grown in a biofilm versus planktonic growth.

| biofilm factor | Gene name or locus tag | product | log2 fold change  (Biofilm vs planktonic cells) |
| --- | --- | --- | --- |
| Intercellular adhesin | *icaA* | poly-beta-1,6 N-acetyl-D-glucosamine synthase | -1.06 |
|  | *icaB* | IcaB | -0.21 |
|  | *icaC* | Polysaccharide intercellular adhesin biosynthesis/export protein IcaC | -0.61 |
|  | *icaD* | intracellular adhesion protein IcaD | -2.40 |
|  | *icaR* | TetR family transcriptional regulator | -0.66 |
| Cell wall-anchored proteins | *sdrG* | fibrinogen-binding adhesin SdrG | 0.43 |
|  | *clfB* | Clumping factor ClfB, fibrinogen binding protein | 0.88 |
|  | *sdrH* | fibrinogen-binding protein | 0.70 |
|  | DMB76_002790 | SDR family oxidoreductase | 2.04 |
|  | DMB76_004160  DMB76_001580 | LPXTG cell wall anchor domain-containing protein | 1.46  2.30 |
|  | *sasD* | cell-wall-anchored protein SasD (LPXAG motif) | 0.66 |
| Biofilm regulation | *srtA* | class A sortase SrtA | 0.70 |
|  | *hlb* | beta-hemolysin: sphingomyelin phosphodiesterase | 1.21 |
|  | DMB76_000415 | beta-class phenol-soluble modulin | 1.54 |
|  | DMB76_009105 | epsilon-class phenol-soluble modulin | 3.39 |
| Autolysin | *atl* | glucosaminidase domain-containing protein | 0.42 |
| Quorum sensing | *agrD* | cyclic lactone autoinducer peptide | 1.60 |
|  | *agrB* | Accessory gene regulator AgrB | 1.21 |
|  | *sarA* | SarA family transcriptional regulator | 1.09 |
