# Supplementary material for: Biofilm formation and inflammatory potential of Staphylococcus saccharolyticus: A possible cause of orthopedic implant-associated infections
Source: Front Microbiol. 2022 Nov 28;13:1070201. doi: 10.3389/fmicb.2022.1070201 (PMC9742538; doi:10.3389/fmicb.2022.1070201)
Supplement: Supplementary file 4 [file Data_Sheet_1.pdf]

## Supplementary figures

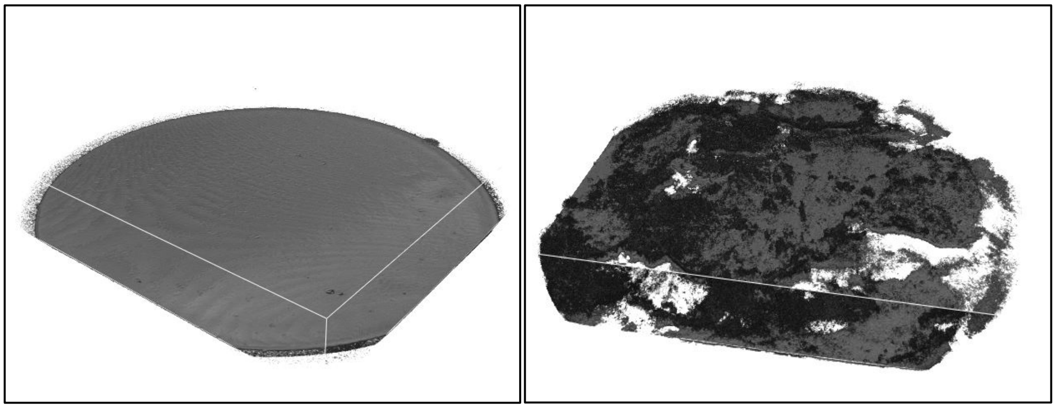

**Figure S1. 3D OCT images of biofilm produced by *S. saccharolyticus* strain 13T0028**. Left, negative control (growth medium (BHCY supplemented with 1% glucose and 1% NaCl) without bacteria); right, biofilm of *S. saccharolyticus* strain 13T0028 grown in growth medium.

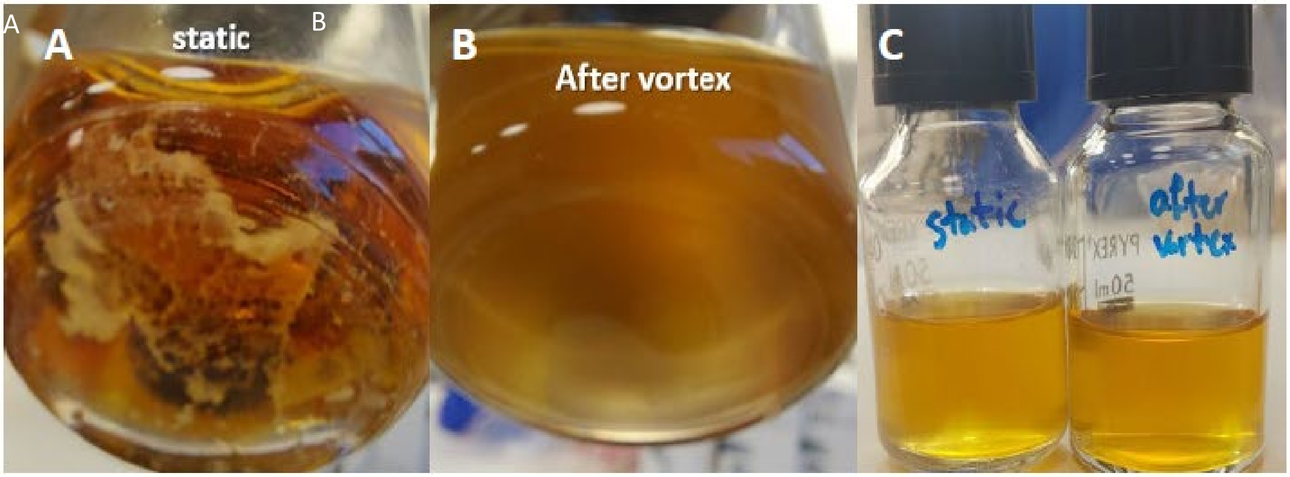


**Figure S2. Macroscopic analysis of autoaggregation of *S. saccharolyticus* 13T0028**. Large aggregation of *S. saccharolyticus* 13T0028 in static culture (A), which can be dissolved after vortexing (B). *S. epidermidis* 1457 remained turbid after 48 h of growth before and after vortexing (C). The media used in the experiment was BHCY supplemented with 1% glucose and 1% NaCl. The experiment was done in triplicates.
